# Visual field loss and vision-related quality of life in the Italian Primary Open Angle Glaucoma Study

**DOI:** 10.1038/s41598-017-19113-z

**Published:** 2018-01-12

**Authors:** Eliana Rulli, Luciano Quaranta, Ivano Riva, Davide Poli, Lital Hollander, Fabio Galli, Andreas Katsanos, Francesco Oddone, Valter Torri, Robert N. Weinreb, Italian Study Group on QoL in Glaucoma, L. Varano, T. Carchedi, S. Talarico, P. Frezzotti, F. Parravano, I. Motolese, S. A. Bagaglia, G. C. M. Rossi, S. Lateri, L. Bossolesi, L. Carmassi, T. Rolle, R. Piccini, R. Ratiglia, A. Rossi, S. Gandolfi, V. Tagliavini, N. Ungaro, M. Fossarello, A. Cucca, I. Zucca, M. Uva, E. Bonacci, G. Cardarella, D. Tognetto, O. Vattovani, P. Vallon, F. Iannacone, L. Fontana, S. Marchi, G. L. Manni, D. Jannetta, G. Roberti, L. Rossetti, E. Maggiolo, O. Oneta, C. Sborgia, F. Cantatore, L. Mastropasqua, L. Agnifili, E. Campos, C. Gizzi, G. Giannaccare, V. Pucci, M. Cassamali, C. Costagliola, C. Traverso, R. Scotto, M. Musolino, L. Landi, A. Bagnis

**Affiliations:** 1 IRCCS - Istituto di Ricerche Farmacologiche Mario Negri, Milan, Italy; 2 DMSS, University of Brescia, Brescia, Italy; 3 IRCCS - Fondazione GB Bietti per lo Studio e la Ricerca in Oftalmologia, Rome, Italy; 4 Hamilton Glaucoma Center, Shiley Eye Institute, and the Department of Ophthalmology, University of California, San Diego, CA USA; 5 Università degli Studi “Magna Graecia”, Catanzaro, Italy; 6 A.O.U. Senese Ospedale Santa Maria delle Scotte, Università di Siena, Siena, Italy; 7 Fondazione IRCCS Istituto di Ricovero e Cura a Carattere Scientifico Policlinico San Matteo, Pavia, Italy; 8 IRCCS Istituto di Ricovero e Cura a Carattere Scientifico Istituto Auxologico Italiano, Milano, Italy; 9 Università degli Studi di Torino, Torino, Italy; 10 Fondazione IRCCS Istituto di Ricovero e Cura a Carattere Scientifico Ca’ Granda Ospedale Maggiore Policlinico, Milano, Italy; 11 A.O.U. di Parma, Parma, Italy; 12 A.O.U. Cagliari - Ospedale Civile San Giovanni di Dio, Cagliari, Italy; 13 A.O.U. “Policlinico Vittorio Emanuele” P.O. Gaspare Rodolico, Catania, Italy; 14 A.O.U. “Ospedale Maggiore”, Trieste, Italy; 15 A.O. Arcispedale Santa Maria Nuova-IRCCS, Reggio Emilia, Italy; 16 Università Tor Vergata, Fondazione Policlinico Tor Vergata, Roma, Italy; 17 A.O. S. Paolo, Milano, Italy; 18 A.O.U. Policlinico, Bari, Italy; 19 Ospedale Clinicizzato SS. Annunziata, Chieti, Italy; 20 A.O.U. Policlinico S. Orsola Malpighi, Bologna, Italy; 21 A.O. di Desenzano del Garda, Desenzano del Garda, Brescia, Italy; 22 Dipartimento SPES, Università del Molise, Campobasso, Italy; 23 IRCCS AOU San Martino - IST, Genova, Italy

**Keywords:** Quality of life, Medical research

## Abstract

The aim of this study was to examine the relationship between visual field (VF) loss, vision-related quality of life (QoL) and glaucoma-related symptoms in a large cohort of primary open angle glaucoma (POAG) patients. POAG patients with or without VF defects or “glaucoma suspect” patients were considered eligible. QoL was assessed using the validated versions of the 25-item National Eye Institute Visual Function Questionnaire (NEI-VFQ-25) and glaucoma-related symptoms were assessed using the Glaucoma Symptom Scale (GSS). Patients were classified as having VF damage in one eye (VFD-1), both eyes (VFD-2), or neither eye (VFD-0). 3227 patients were enrolled and 2940 were eligible for the analysis. 13.4% of patients were classified in the VFD-0, 23.7% in the VFD-1, and 62.9% in the VFD-2 group. GSS visual symptoms domain (Func-4) and GSS non-visual symptoms domain (Symp-6) scores were similar for the VFD-0 and VFD-1 groups (p = 0.133 and p = 0.834 for Func-4 and Symp-6, respectively). VFD-0 group had higher scores than VFD-2 both in Func-4 (p < 0.001) and Symp-6 domains (p = 0.035). Regarding the NEI-VFQ-25, our data demonstrated that bilateral VF defects are associated with vision-related QoL deterioration, irrespective of visual acuity.

## Introduction

Glaucoma constitutes a major global cause or irreversible visual loss^1^. It is estimated that approximately 60 million people worldwide have glaucoma and 8.4 million patients are bilaterally blind^1^. Of the many types of the disease, primary open angle glaucoma (POAG) is by far the commonest in populations of European origin^2,3^. As a chronic, progressive, vision-threatening disease, POAG can severely affect vision-related quality of life (QoL). Visual decline is a direct consequence of the glaucomatous process and can lead to limitations of daily functioning and loss of autonomy, thus causing deterioration of vision-related QoL and significant psychological burden^4,5^. In addition to the degradation of QoL due to the disease-related visual decline, factors such as adverse events of medication, cost of treatment, or even the distress elicited by the mere diagnosis of an irreversible, potentially blinding disorder can adversely affect the patient’s sense of well-being and QoL^6–13^.

The importance of preserving vision-related QoL at a sustainable cost has become increasingly recognized in glaucoma management^14^. Evidence has shown that patients with glaucoma often have problems with important daily activities such as walking^15^, driving^16–18^, or reading^18^, especially when perimetric damage is advanced or when both eyes are affected. Despite the recent interest in glaucoma-related QoL issues^13,19–22^, the relationship between vision-specific QoL and severity of visual field (VF) defects or number of eyes with perimetric loss warrants further exploration in sufficiently powered studies. In addition, the relationship between severity of VF damage or number of perimetrically affected eyes and glaucoma-related visual and non-visual symptoms needs to be better characterized in adequately sized studies.

In a previous paper of ours^23^, we described the methodology of the Italian Primary Open Angle Glaucoma Study (IPOAGS) and the baseline characteristics of the participants. In addition, that paper reported the association between vision-related QoL assessed with the 25-item National Eye Institute Visual Function Questionnaire (NEI-VFQ-25) and glaucoma-related symptoms assessed with the Glaucoma Symptoms Scale (GSS) questionnaire^23^. It also presented the association between the NEI-VFQ-25 and GSS scores with glaucoma severity in a sample of 3169 patients^23^. In the current paper, our analysis was primarily focused on the impact of glaucoma (in terms of vision-related QoL and glaucoma-related symptoms) according to the number of perimetrically affected eyes. Therefore, the aim of the current report is to describe the characteristics of IPOAGS patients who have no-, one-, or both eyes with visual field loss, and examine the association between the number of perimetrically affected eyes and NEI-VFQ-25 and GSS scores.

## Patients and Methods

Details regarding the multicenter IPOAGS study have been described elsewhere^23^. Briefly, twenty-one academic and non-academic Italian institutions were involved in the recruitment. Patients aged >18 years with a previous or new diagnosis (or strong clinical suspicion) of POAG were invited to participate during regular visits and were consecutively enrolled. Patients were considered eligible for inclusion if they had optic nerve head damage typical for glaucoma, i.e. focal and/or generalized neuroretinal rim thinning or cup/disc ratio asymmetry >0.2 in the absence of other neurodegenerative diseases. The presence of characteristic glaucomatous visual field defects was not a prerequisite for inclusion. Women who were pregnant, breast-feeding or had plans to become pregnant were excluded. Other exclusion criteria were the concurrent abuse of alcohol or illicit substances, and the participation in any clinical trial that tested medications or medical devices within 30 days prior to recruitment. The validated Italian version of the NEI-VFQ-25 was used for the assessment of vision-related QoL^24^. The validated Italian version of the GSS Questionnaire^25^ was used for the assessment of glaucoma-related visual and non-visual symptoms.

The NEI-VFQ-25 is a self-administered 25-item, 12-subscale questionnaire assessing the following: general health, general vision, ocular pain, near activities, distant activities, social functioning, mental health, role difficulties, dependency, driving, color vision, and peripheral vision^26–28^. The scoring procedure converts the pre-coded numeric values of each item to a score ranging from 0 to 100. Higher scores reflect better vision-related QoL. The global score is the mean score of all items that constitute 11 of the 12 subscales, with the exception of the single item that constitutes the general-health subscale.

The GSS Questionnaire^29^, a glaucoma specific tool, is a modified version of a checklist of symptoms developed by the Ocular Hypertension Treatment Study group. The tool includes 10 items grouped into two domains: Symp-6 for non-visual symptoms (burning/smarting/stinging, tearing, dryness, itching, soreness/tiredness, feeling of something in the eye) and Func-4 for visual symptoms (blurry/dim vision, hard to see in daylight, hard to see in darkness, and halos around lights). The ten items assess each eye separately using a scale from 0 to 100, where zero indicates the presence of a very bothersome symptom and 100 the absence of the symptom. The domain score is the un-weighted average of the scores of the items that make up the domain in question. Similarly, the total GSS score is the mean of the 10 item scores. GSS scores can be calculated as average of the two eyes and for each eye separately.

**Figure 1:**
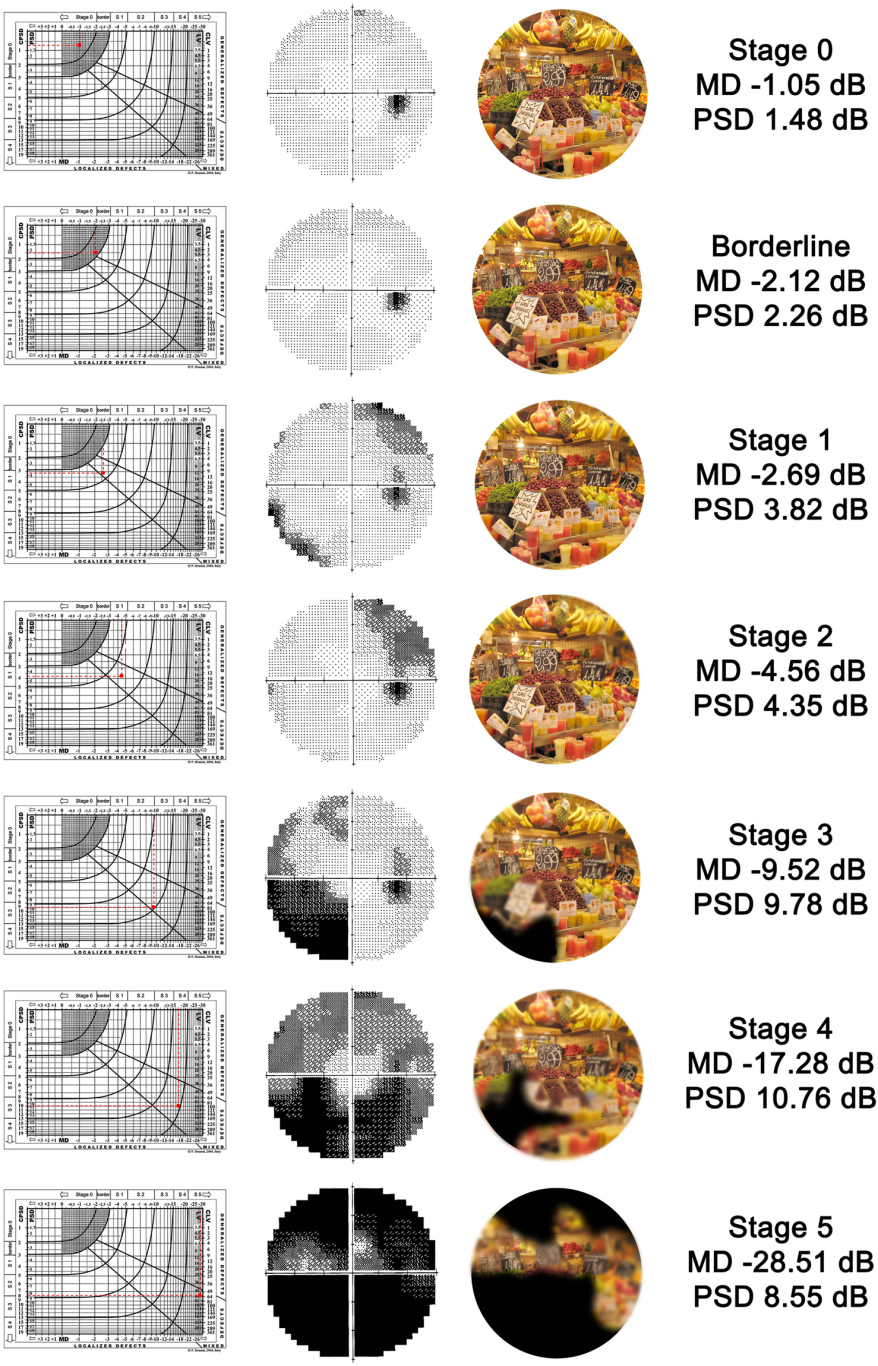
Visual field stages according to Glaucoma Staging System 2: simulation of what patients see at different stages. On the left Glaucoma Staging System 2 diagrams, in the middle visual field gray scale, on the right simulation of what patients see.

The severity of VF damage was classified according to the Glaucoma Staging System 2^30–32^. The Glaucoma Staging System 2 is a classification method^31^ that uses Mean Defect or Mean Deviation (MD) and Pattern Standard Deviation/Corrected Pattern Standard Deviation (PSD/CPSD) or Loss Variance/Corrected Loss Variance (LV/CLV) values (from either the 30-2/24-2 Zeiss-Humphrey tests or the G1/G1X/G2 Octopus programs) on a Cartesian coordinate diagram. The visual fields are divided in seven different stages by curvilinear lines from stage 0 (normal visual fields) through borderline to stage 5 (low threshold readings, with only small remnants of sensitivity remaining). This nomogram allows the user to quickly determine the stage of the disease (Fig. 1). When compared to other VF staging systems (e.g. The Advanced Glaucoma Intervention Study scoring system^33^ or the Hodapp-Anderson-Parrish system^34^), the Glaucoma Staging System 2 has been demonstrated to be preferable for its ease of use for clinicians and researchers alike^35^. Furthermore, it has been used in population-based studies and clinical trials^36–40^.

Based on the presence of glaucoma and perimetric damage, eyes were classified in three categories: a) eyes without signs of glaucoma (WOG), i.e. fellow eyes of patients with unilateral glaucoma; b) eyes with glaucoma but no VF damage (WOD), i.e. “pre-perimetric” glaucoma: VF stage 0-borderline; c) eyes with glaucoma and VF damage (WD; VF stage 1 or greater). Participants, on the other hand, were classified in three categories: patients without VF damage in any eye (VFD-0), patients with VF damage in one eye (VFD-1), and patients with VF damage in both eyes (VFD-2). Two analyses were performed, one considering patients as units of analysis, and one considering eyes as units of analysis.

Descriptive summary statistics are presented as mean, standard deviation, minimum and maximum values for continuous variables, and as absolute frequency and percentage for categorical variables. The Chi-square test for trend and linear regression were used to investigate associations between VF and clinical characteristics. When considering patients as units of analysis a linear regression model was fitted to test the effect of number of eyes with VF damage on questionnaires scores (performed with a generalized linear model procedure). For each subscale, as well as for the total score of both the NEI-VFQ-25 and the GSS questionnaires, a multivariate model was fitted with the following covariates: age, best corrected visual acuity (BCVA, mean of the two eyes), previous treatment (treated versus untreated) and number of eyes with VF damage (VFD-0, VFD-1, VFD-2). VFD-0 was chosen as reference group for the latter variable; therefore p-values for VFD-1 and VFD-2 refer to the comparison of these groups versus VFD-0. For the “previous treatment” variable, untreated previously diagnosed or newly diagnosed patients were considered “untreated”, while previously diagnosed patients who had received medical and/or surgical treatment (or were currently under medical treatment) were considered “treated”.

When considering eyes as units of analysis, since we had to consider more than one measurement on the same subject (VF stage according to the Glaucoma Staging System 2, BCVA, MD, PSD, GSS questionnaire score), we needed to consider the correlation between these variables on the same subject. This analysis was performed with a mixed model in order to take into account the correlation between eyes of the same subject (performed with mixed procedure). In our analysis the covariance structure hypothesized was the unstructured matrix. “Unstructured” means that no constraints were imposed on the values of covariance and variance. Therefore, the model estimates these quantities. Because there are about 6000 observations in our analysis, our study had the statistical power necessary to estimate the covariance structure from the data without compromising the efficiency of the model.

Two multivariate analyses were performed to test the effect of presence and stage of VF damage at eye level on GSS questionnaire scores: the covariates of the first analysis included eye groups according to presence of glaucoma and VF damage (WOG, WOD, WD), age, BCVA and previous treatment (treated versus untreated). The covariates of the second analysis included VF stage (according to the Glaucoma Staging System 2), age and BCVA. All analyses were performed with the SAS software (version 9.2).

The study was conducted in accordance with the tenets of the Declaration of Helsinki and the Guidelines for Good Clinical Practice. The ethic committee of Brescia (Comitato Etico provinciale di Brescia), and of each participating centre approved the protocol (see list of Membership of the Italian Study Group on QoL in Glaucoma and participating centers). For inclusion, patients had to provide informed consent after the nature and purpose of the survey were fully explained. The study was registered with clinicaltrials.gov (NCT01742104).

## Data Availability

The datasets generated during the current study are available from the corresponding author on reasonable request.

## Results

From March 2012 to July 2013, 3227 patients were enrolled in 21 centers. Two hundred eighty seven patients were subsequently excluded due to incomplete filling of questionnaires (n = 58), incomplete data on staging (n = 47) or missing data on BCVA (n = 182). Consequently, 2940 patients were included in the per-eye analysis. As data on both eyes was not available for all patients, 2823 patients were included in the per-patient analysis. The demographic characteristics of the participants are presented in Table 1.

**Table 1:** Characteristics of study subjects based on the number of eyes with visual field damage.

	VFD-0 (n = 378)	VFD-1 (n = 689)	VFD-2 (n = 1776)	p-value
***Age (years)***				
Mean (SD)	62.4 (11.4)	65.0 (11.8)	68.8 (11.7)	<0.001*
Min-max	23.6–91.1	23.8–100.0	18.6–100.6	
***Male gender***	186 (49.2)	338 (50.5)	876 (49.3)	0.863^
***Race***				
Asian	0 (0.0)	2 (0.3)	3 (0.2)	0.876^
Black	1 (0.3)	4 (0.6)	9 (0.5)	
Caucasian	377 (99.7)	663 (99.1)	1764 (99.3)	
***BCVA (Decimal fraction) [Snellen]***				
Mean (SD)	0.93 (0.1) [20/21.5 (20/200)]	0.87 (0.2) [20/23.0 (20/100)]	0.79 (0.2) [20/25.3 (20/100)]	<0.001*
Min-max	0.04–1.0 [20/500–20/20]	0.02–1.0 [20/1000–20/20]	0.00–1.0 [0.0 –20/20]	
**Newly Diagnosed**	20 (5.29)	39 (5.83)	108 (6.08)	0.557^
***Previous treatment#***				<0.001^
None	22 (6.2)	21 (3.3)	27 (1.6)	
Only medical	279 (77.9)	419 (66.5)	1194 (71.6)	
Only surgical	8 (2.2)	20 (3.2)	60 (3.6)	
Medical and surgical	49 (13.7)	170 (27.0)	386 (23.2)	
***Type of surgery (n = 693)***				
Trabeculectomy	21 (36.8)	95 (50.0)	268 (60.1)	<0.001^
Viscocanalostomy	0 (0.0)	2 (1.1)	1 (0.2)	0.537^
Deep-sclerectomy	0 (0.0)	3 (1.6)	16 (3.6)	0.053^
Argon laser trabeculoplasty	40 (70.2)	117 (61.6)	205 (46.0)	<0.001^
***Glaucoma medication***	328 (91.6)	589 (93.5)	1581 (94.8)	0.017^
β-blockers	93 (28.4)	152 (25.9)	364 (23.0)	0.025^
CAIs	22 (6.7)	57 (9.7)	214 (13.5)	<0.001^
Prostaglandin analogues	167 (50.9)	275 (46.7)	830 (52.5)	0.163^
Parasympathomimetic drugs	5 (1.5)	28 (4.8)	53 (3.4)	0.456^
Prostaglandin + β-blocker FC	47 (14.3)	127 (21.6)	327 (20.7)	0.045^
Alpha agonist + β-blocker FC	10 (3.1)	30 (5.1)	72 (4.6)	0.432^
CAI + β-blocker FC	56 (17.1)	129 (21.9)	416 (26.3)	<0.001^
***Systemic treatment***	234 (61.9)	430 (64.5)	1299 (73.5)	<0.001^
***Glaucoma family history***	154 (40.7)	242 (36.2)	614 (34.6)	0.029^
***Myopia***	49 (13.0)	112 (16.7)	327 (18.4)	0.012^
***Diabetes***	40 (10.6)	75 (11.2)	287 (16.2)	<0.001^
***Hypertension***	186 (49.2)	331 (49.5)	998 (56.2)	0.001^

Min, minimum value; max, maximum value; SD, standard deviation; BCVA, best corrected visual acuity; VFD-0, patients without visual field damage in any eye; VFD-1, patients with visual field damage in one eye; VFD-2, patients with visual field damage in both eyes; CAI, carbonic anhydrase inhibitor; FC, fixed combination; *, linear regression model; ^, chi square test for trend; ^#^in patients not newly diagnosed.

### Patient as unit of analysis

Three hundred seventy eight patients (13.4%) were grouped as VFD-0, 669 (23.7%) as VFD-1 and 1776 (62.9%) as VFD-2. Having more eyes with VF damage was associated with older age (p < 0.001) and worse BCVA (p < 0.001), but not with gender and race. Family history (p = 0.029), myopia (p = 0.012), diabetes (p < 0.001) and hypertension (p = 0.001) were all risk factors for having bilateral VF damage.

The percentage of patients using glaucoma medications was increasingly higher as the number of eyes with VF defects increased (91.6% for VFD-0 vs 93.5% for VFD-1 vs 94.8% for VFD-2, p = 0.017). Similarly, the percentage of patients using systemic concomitant treatments was increasingly higher as the number of eyes with VF defects increased (61.9% for VFD-0 vs 64.5% for VFD-1 vs 73.5% for VFD-2, p < 0.001) (Table 1).

Descriptive statistics for the GSS and NEI-VFQ-25 questionnaires are reported in Table 2. For the GSS questionnaire, the score of the visual symptoms domain (Func-4) for VFD-0 was similar to VFD-1 (adjusted mean difference (aMD) = −2.16 standard error (SE) = 1.44,p = 0.133), but significantly higher than VFD-2 patients (aMD = −8.30, SE = 1.30, p < 0.001) after adjusting for age, BCVA and previous treatment. The score of the non-visual symptoms domain (Symp-6) for VFD-0 was similar to VFD-1 (aMD = −0.29, SE = 1.36, p = 0.834), but higher than VFD-2 (aMD = −2.61, SE = 1.24, p = 0.035) after adjusting for age, BCVA and previous treatment. Better BCVA and advanced age were associated with higher scores for both domains (BCVA: p < 0.001 and p = 0.012 for Func-4 and Symp-6 respectively; age: p < 0.001 and 0.032 for Func-4 and Symp-6 respectively), while previous treatment was not associated with any score (p = 0.063 and p = 0.276 for Func-4 and Symp-6 respectively). The same pattern was observed for the total score (Table 3).

**Table 2:** Glaucoma Symptom Scale (GSS) and National Eye Institute Visual Function (NEI-VFQ-25) questionnaire scores based on the number of eyes with visual field damage.

	VFD-0	VFD-1	VFD-2
	Mean (SD)	Mean (SD)	Mean (SD)
**GSS questionnaire**			
Func-4	84.0 (18.6)	80.5 (19.7)	73.0 (24.6)
Symp-6	76.1 (21.7)	75.6 (20.2)	73.2 (21.3)
Total score	79.4 (18.0)	77.6 (17.3)	73.1 (19.7)
**NEI-VFQ-25**			
General health	60.88 (17.04)	58.77 (17.53)	55.25 (17.82)
General vision	68.06 (13.16)	64.31 (14.42)	58.05 (16.21)
Ocular pain	81.3 (18.6)	79.28 (18.57)	76.83 (20.72)
Near activities	90 (13.46)	86.41 (15.68)	79.97 (20.9)
Distance activities	94.74 (9.23)	91.48 (13.12)	85.88 (18.44)
Vision-specific social functioning	98.18 (6.94)	96.72 (9.61)	93.18 (14.52)
Vision-specific mental health	84.83 (14.09)	81.09 (17.65)	75.9 (21.35)
Vision-specific role difficulties	95.02 (12.41)	91.73 (15.64)	86.26 (21.05)
Vision-specific dependency	97.64 (8.91)	95.44 (13.88)	90.57 (20.14)
Driving	90.38 (14.49)	83.91 (20.99)	78.61 (25.26)
Color vision	98.67 (7.62)	96.95 (11.28)	93.82 (16.38)
Peripheral vision	94.69 (12.57)	89.46 (18.33)	83.7 (23.01)
Total score	90.27 (8.22)	87.00 (11.29)	82.03 (15.74)

SD, standard deviation; VFD-0, patients without visual field damage in any eye; VFD-1, patients with visual field damage in one eye; VFD-2, patients with visual field damage in both eyes; GSS, Glaucoma Symptom Scale; NEI-VFQ-25, National Eye Institute Visual Function Questionnaire-25.

**Table 3:** Glaucoma Symptom Scale (GSS) questionnaire scores and number of eyes with visual field damage: statistical analysis (per-patient analysis).

	Intercept		VFD-1		VFD-2		Age		BCVA		Previous treatment	
	β (Stderr)	p-value	β (Stderr)	p-value*	β (Stderr)	p-value*	β (Stderr)	p-value	β (Stderr)	p-value	β (Stderr)	p-value
Func-4	54.24 (3.69)	<0.001	−2.16 (1.44)	0.133	−8.3 (1.3)	<0.001	0.13 (0.04)	<0.001	25.75 (2.11)	<0.001	−2.82 (1.52)	0.063
Symp-6	68.14 (3.51)	<0.001	−0.29 (1.36)	0.834	−2.61 (1.24)	0.035	0.07 (0.03)	0.032	5.04 (2.01)	0.012	−1.57 (1.44)	0.276
*Total score*	*62.58 (3.12)*	<0.001	−*1.15 (1.21)*	0.344	−*4.97 (1.1)*	<0.001	*0.1 (0.03)*	*0*.001	*13.32 (1.78)*	<0.001	−*1.98 (1.28)*	*0.123*

VFD-1, patients with visual field damage in one eye; VFD-2, patients with visual field damage in both eyes; BCVA, best corrected visual acuity; β, beta coefficient; SE, standard error for beta coefficient; p-value for multivariate linear regression model including visual field damage groups, BCVA, age, previous treatment (treated versus untreated); *, p-values for VFD-1 and VFD-2 refer to the comparison between each group and VFD-0 (patients without visual field damage in any eye).

Regarding the NEI-VFQ-25 questionnaire, our data indicate that bilateral VF defects are associated with vision-related QoL deterioration irrespective of BCVA (Table 4). Additionally, patients in the VFD-0 group had significantly higher vision-related QoL scores than patients in the VFD-1 group in the “General vision” (p = 0.029), “Driving” (p = 0.009) and “Peripheral vision” (0.013) subscales after adjusting for age, BCVA and previous treatment. In other words, even patients with VF defects in only one eye had significantly worse vision-related QoL scores in 3 of 12 subscales compared to patients without VF defects in any eye. Better BCVA was associated with higher scores for all subscales (p < 0.001 for all subscales). A similar pattern was observed for the total NEI-VFQ-25 score (Table 4).

**Table 4:** National Eye Institute Visual Function (NEI-VFQ-25) questionnaire scores and number of eyes with visual field damage: statistical analysis (per-patient analysis).

	Intercept		VFD-1		VFD-2		Age		BCVA		Previous treatment	
	β (Stderr)	p-value	β (Stderr)	p-value*	β (Stderr)	p-value*	β (Stderr)	p-value	β (Stderr)	p-value	β (Stderr)	p-value
General health	67.99 (2.91)	<0.001	−1.25 (1.13)	0.268	−3.76 (1.02)	<0.001	−0.16 (0.03)	<0.001	5.59 (1.67)	<0.001	−2.45 (1.20)	0.041
General vision	47.98 (2.42)	<0.001	−2.06 (0.94)	0.029	−6.49 (0.85)	<0.001	0 (0.02)	0.973	25.04 (1.39)	<0.001	−3.53 (1.00)	<0.001
Ocular pain	68.37 (3.29)	<0.001	−1.36 (1.28)	0.289	−3.13 (1.16)	0.007	0.08 (0.03)	0.021	12.47 (1.89)	<0.001	−3.81 (1.36)	0.005
Near activities	63.44 (2.97)	<0.001	−1.59 (1.16)	0.170	−5.85 (1.05)	<0.001	0 (0.03)	0.876	30.53 (1.7)	<0.001	−2.36 (1.22)	0.054
Distance activities	69.37 (2.53)	<0.001	−1.36 (0.99)	0.169	−4.84 (0.89)	<0.001	−0.01 (0.03)	0.737	29.03 (1.45)	<0.001	−1.21 (1.04)	0.244
Social functioning	82.18 (2.00)	<0.001	−0.09 (0.78)	0.907	−2.12 (0.71)	0.003	−0.03 (0.02)	0.196	19.83 (1.15)	<0.001	−0.94 (0.82)	0.255
Mental health	58.59 (3.14)	<0.001	−2.08 (1.22)	0.088	−5.57 (1.11)	<0.001	0.08 (0.03)	0.009	27.42 (1.8)	<0.001	−4.9 (1.29)	<0.001
Role difficulties	68.99 (2.98)	<0.001	−1.32 (1.16)	0.257	−4.65 (1.05)	<0.001	0.01 (0.03)	0.665	30.08 (1.71)	<0.001	−3.07 (1.23)	0.012
Dependency	77.4 (2.8)	<0.001	−0.4 (1.09)	0.714	−3.26 (0.99)	0.001	−0.05 (0.03)	0.104	25.8 (1.61)	<0.001	−1.03 (1.15)	0.370
Driving	67.31 (4.23)	<0.001	−4.1 (1.56)	0.009	−7.29 (1.42)	<0.001	−0.08 (0.04)	0.043	32.7 (2.55)	<0.001	−2.56 (1.76)	0.146
Color vision	85.18 (2.32)	<0.001	−0.42 (0.9)	0.642	−2.12 (0.81)	0.009	−0.04 (0.02)	0.110	18.23 (1.33)	<0.001	−1.3 (0.95)	0.172
Peripheral vision	68.79 (3.31)	<0.001	−3.18 (1.29)	0.013	−6.7 (1.16)	<0.001	0 (0.03)	0.971	30.93 (1.89)	<0.001	−3.11 (1.36)	0.022
*Total score*	68.69 (2.16)	<0.001	−1.58 (0.84)	0.060	−4.7 (0.76)	<0.001	0 (0.02)	0.944	25.57 (1.24)	<0.001	−2.56 (0.89)	0.004

SD, standard deviation; VFD-1, patients with visual field damage in one eye; VFD-2, patients with visual field damage in both eyes; BCVA, best corrected visual acuity; β, beta coefficient; SE, standard error for beta coefficient; p-value for multivariate linear regression model including visual field damage groups, BCVA, age, previous treatment (treated versus untreated); *, p-values for VFD-1 and VFD-2 refer to the comparison between each group and VFD-0 (patients without visual field damage in any eye).

### Eye as unit of analysis

When considering eyes as units of analysis, 106 (1.9%) out of 5729 eyes were classified as WOG, 1304 (22.8%) as WOD and 4319 (75.4%) as WD.

**Figure 2:**
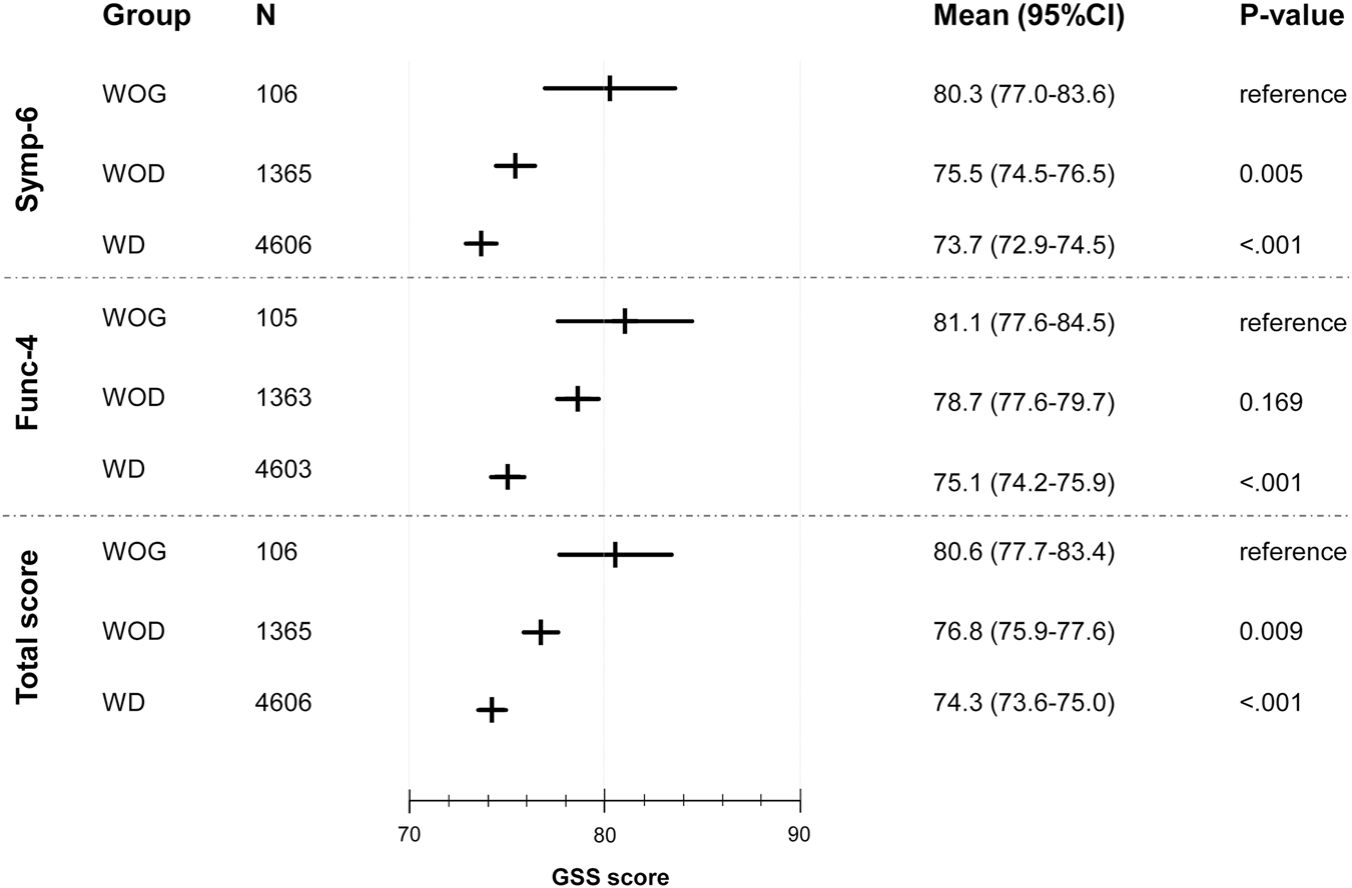
Glaucoma Symptom Scale (GSS) questionnaire scores depending on the presence of glaucoma and visual field damage (eye as unit of analysis).

Results of the GSS questionnaire based on presence of glaucoma and VF loss are depicted in Fig. 2. After adjusting for age, BCVA and previous treatment, not only glaucomatous eyes with VF defects (WD), but also glaucomatous eyes without VF defects (WOD) were associated with lower scores in the non-visual symptoms domain (Symp-6) compared to eyes without glaucoma (WOG) (p = 0.005 and p < 0.001 for WOD and WD respectively). For the visual symptoms domain (Func-4) only WD showed a lower score than eyes without glaucoma (WOG) (p < 0.001), while no statistically significant difference was detected between WOD and WOG (p = 0.169) (Table 5). Better BCVA was associated with higher score for both domains and total score (p < 0.001). Age was statistically significant only for Symp-6 (p = 0.04), while previous treatment was only significant for Func-4 (p = 0.013).

**Table 5:** Glaucoma Symptom Scale (GSS) questionnaire scores and visual field damage: statistical analysis (per-eye analysis).

	Func-4		Symp-6		Total score	
	β (Stderr)	p-value	β (Stderr)	p-value	β (Stderr)	p-value
Intercept	69.55 (2.81)	<0.001	66.89 (2.61)	<0.001	*67.9 (2.33)*	**<*0.001***
WOD*	−2.41 (1.75)	0.169	−4.84 (1.7)	0.005	−*3.81 (1.47)*	*0.009*
WD*	−6 (1.71)	<0.001	−6.59 (1.66)	<0.001	−*6.31 (1.43)*	**<*0.001***
Age	0.02 (0.04)	0.555	0.07 (0.03)	0.040	*0.05 (0.03)*	*0.100*
BCVA	9.27 (0.77)	<0.001	4.37 (0.74)	<0.001	*6.34 (0.64)*	**<*0.001***
Previous treatment	−3.89 (1.56)	0.013	−1.48 (1.44)	0.306	−*2.34 (1.29)*	*0.070*

WOD, eyes with glaucoma but no visual field damage; WD, eyes with glaucoma and visual field damage (stage 1 or greater); BCVA, best corrected visual acuity; β, beta coefficient; SE, standard error for beta coefficient; p-value for multivariate mixed model including visual field damage groups, BCVA, age, previous treatment (treated versus untreated); *, p-values for WOD and WD refer to the comparison between each group and the WOG (eyes without signs of glaucoma).

**Figure 3:**
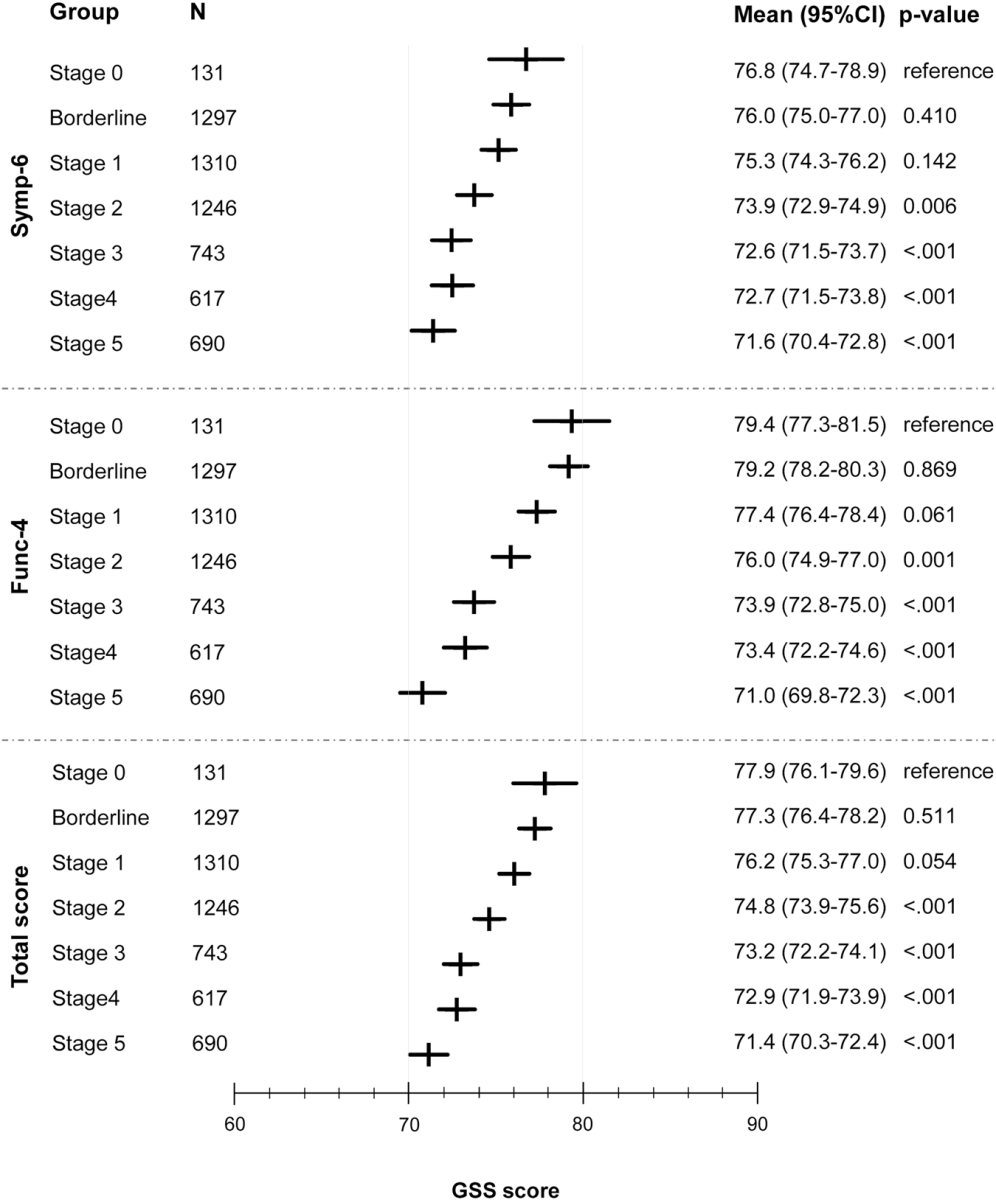
Glaucoma Symptom Scale (GSS) questionnaire scores by stage according to Glaucoma Staging System 2 (eye as unit of analysis).

The analysis of GSS results based on stage as determined with Glaucoma Staging System 2 is depicted in Fig. 3. Both in the non-visual symptoms domain (Symp-6) and in the visual symptoms domain (Func-4), a significant inverse relationship between VF stage and GSS score was seen after adjustment for age, BCVA and previous treatment. The differences became statistically significant at stage 2, compared to stage 0. The same association was observed for total score (Table 6). Better BCVA was associated with higher score for both domains and total score. With the exception of the non-visual symptoms domain (Symp-6) (p = 0.025), age was not statistically significant. Previous treatment was statistically significant only for Func-4 (p = 0.025).

**Table 6:** Glaucoma Symptom Scale (GSS) questionnaire scores and stage of visual field damage: statistical analysis (per-eye analysis).

	Func-4		Symp-6		Total score	
	β (Stderr)	p-value	β (Stderr)	p-value	β (Stderr)	p-value
Intercept	66.96 (2.81)	<0.001	65.5 (2.63)	<0.001	66.01 (2.34)	<0.001
Borderline*	−0.17 (1)	0.869	−0.82 (1)	0.410	−0.56 (0.85)	0.511
Stage 1*	−1.97 (1.05)	0.061	−1.53 (1.04)	0.142	−1.71 (0.89)	0.054
Stage 2*	−3.42 (1.07)	0.001	−2.89 (1.06)	0.006	−3.11 (0.9)	<0.001
Stage 3*	−5.48 (1.1)	<0.001	−4.18 (1.09)	<0.001	−4.72 (0.93)	<0.001
Stage 4*	−5.97 (1.12)	<0.001	−4.12 (1.11)	<0.001	−4.93 (0.95)	<0.001
Stage 5*	−8.36 (1.15)	<0.001	−5.2 (1.14)	<0.001	−6.5 (0.97)	<0.001
Age	0.03 (0.04)	0.422	0.07 (0.03)	0.025	0.06 (0.03)	0.060
BCVA	6.46 (0.81)	<0.001	2.77 (0.79)	<0.001	4.24 (0.68)	<0.001
Previous treatment	−3.5 (1.56)	0.025	−1.28 (1.45)	0.377	−2.07 (1.29)	0.110

BCVA, best corrected visual acuity; β, beta coefficient; SE, standard error for beta coefficient; p-value for multivariate mixed model including visual field stage groups, BCVA, age, previous treatment (treated versus untreated); *, p-values for borderline, stage1, stage 2, stage 3, stage 4, stage 5 groups refer to the comparison between each stage group and stage 0.

## Discussion

In the current study, being older was associated with a higher number of perimetrically affected eyes. A possible explanation for this association is that glaucoma is a chronic disease of the elderly, and the chances of perimetric defects appearing in one or both eyes increase with advancing age. Moreover, we found that factors such as family history, myopia, diabetes and systemic hypertension were associated with bilateral VF damage. These conditions have not consistently been demonstrated to be risk factors for the onset and progression of the disease^41,42^.

The proportion of patients using concomitant systemic treatments was higher for those with VF damage. Evidence has shown that systemic medications such as statins^43,44^, calcium channel blockers^45^ or diuretics^46^ have an influence on glaucoma risk. The exact effect of common systemic vasoactive medications on glaucoma risk remains to be determined. In the case of systemically administered β-blockers for instance, although this class of medications can have a protective effect in glaucoma because of a certain ocular hypotensive effect, the concurrent reduction in blood pressure may compromise optic nerve perfusion pressure^47^. In fact, evidence from population-based studies has shown that systemic hypotension may be associated with an increased prevalence and incidence of open-angle glaucoma^48–50^.

Deterioration of vision-related QoL has been reported even in patients with early VF loss^21,22^. In the Los Angeles Latino Eye Study for instance, adults with glaucoma experienced measurable loss in QoL early in the disease process^22^. Our data from a larger, ethnically different population are in accordance with those results: we found that patients with VF damage in one eye had lower vision-related QoL scores in three of twelve NEI-VFQ-25 subscales compared to patients without VF damage in any eye. There are several reasons for the deterioration of QoL in early glaucoma. The most important is obviously related to the adverse effects, inconvenience and cost of antiglaucoma medications^51–53^. Another reason may be the psychological burden of suffering from a potentially blinding disease^54^. This psychological pressure may explain the observation that even the diagnosis of “glaucoma suspect” is associated with deterioration of vision-related QoL^9,55,56^. At least in theory, a third reason that could explain the deterioration of vision-related QoL even in perimetrically unaffected glaucomatous eyes is that certain aspects of visual function beyond retinal sensitivity, such as color perception, contrast sensitivity and motion perception are affected early in the glaucomatous process^57,58^.

In our study, both visual and non-visual glaucoma-related symptoms were more bothersome with increasing stage of perimetric damage. Of note, eyes with pre-perimetric glaucoma (or eyes suspicious for glaucoma) had worse score in the non-visual symptoms domain and worse total score at the GSS questionnaire than eyes without glaucoma. We believe that the worse symptom scores in these eyes are due to the use of anti-glaucoma medications.

In the present investigation, patients with glaucoma and VF damage in both eyes had significantly worse scores in both the non-visual and the visual symptom domains of the GSS tool compared to patients without VF defects in any eye. The visual symptoms domain in particular showed good discrimination between patients with visual field defects in both eyes and patients without visual field defects in any eye. In patients with advanced glaucoma, the areas of VF defects in each eye may coincide, resulting in binocular VF loss^59^. Several studies have shown that patients with binocular VF loss experience severe difficulties in activities of daily life, such as reading, moving around or driving^15–18^. The location of VF defects may also play an important role in the patients’ functioning and perception of vision-related QoL^22^. For example, Sawada *et al*.^60^ have shown that perimetric defects in the lower paracentral visual field of the better eye have the strongest correlation with NEI-VFQ-25 scores. These authors also reported that defects in the upper temporal visual field have a strong impact on the driving subscale of the NEI-VFQ-25 questionnaire (*r* = 0.509, p < 0.001), while defects at the lower peripheral visual field strongly correlate with subscales such as role limitation (*r* = 0.459, p < 0.001) and peripheral vision (*r* = 0.425, p < 0.001)^60^. Other investigators have found that superior perimetric defects in binocular integrated visual fields are associated with difficulty with near activities, while inferior perimetric defects in binocular integrated visual fields are associated with vision-specific role difficulties, as well as general and peripheral vision^61^.

Our report provides evidence that VF loss is associated with decreased vision-related QoL in a manner that is independent of BCVA deterioration. To date, several studies have shown that visual acuity loss is one of the causes associated with lower vision-related QoL in glaucoma patients^62–64^.

The current study constitutes the largest investigation on vision-related QoL and glaucoma-related symptoms^12,22,24,25,28,65–80^. However, our sample cannot be considered representative in a strict methodological sense because it was not formed by means of random inclusion from a central nationwide registry. Since such a registry is not available, our sample can be considered as close as possible to being representative by virtue of its wide-ranging geographical distribution, recruitment from diverse academic and non-academic centers, and size.

Another limitation of our study is related to the use of the NEI-VFQ-25 questionnaire. Despite its widespread adoption in QoL research, this tool is not free of drawbacks. Although traditional validation techniques have shown that the tool is valid and reliable for the evaluation of vision-specific QoL, some advanced statistical approaches have detected low precision at least for some of its items^67,73,81,82^. In general, the evaluation of QoL using a questionnaire has some limitations. One of them is that QoL assessment is subjective, so that patients with similar disability may rate their QoL differently. Another inherent limitation of this type of investigations is that self-reported visual ability can be impaired, at least to some extent, by several ophthalmic and systemic comorbidities and psychosocial constraints. Conceivably, even when VF indices such as MD are comparable, a multitude of diverse determinants such as spatial distribution and depth of VF scotomas or speed of VF deterioration may affect differently patients with dissimilar lifestyles and expectations^83^. A certain limitation of our eye-level analysis is that the “non-glaucomatous” eyes (WOG) were not eyes of healthy controls, but fellow eyes of patients with monocular glaucoma (or fellow eyes of patients with high suspicion for monocular glaucoma.

In conclusion, in the current study, having more eyes with VF damage was associated with older age and worse BCVA. Self-reported family history of glaucoma, myopia, diabetes mellitus and systemic hypertension were all associated with bilateral VF defects. The percentage of patients using glaucoma medications was increasingly higher as the number of eyes with VF defects increased. At the patient level analysis, participants with no VF defects in any eye or VF defects in one eye had significantly better scores in the glaucoma-related vision- and non-vision symptom scores of the GSS instrument compared to patients with bilateral VF defects after adjusting for age and BCVA. After adjusting for age and BCVA, patients without VF defects in any eye had significantly better NEI-VFQ-25 scores in 3 of 12 subscales (“general vision”, “driving”, “peripheral vision”) compared to patients with VF defects in one eye, and better NEI-VFQ-25 scores in all subscales compared to patients with VF defects in both eyes. At the eye level analysis, after adjusting for age and BCVA, eyes without glaucoma had significantly better scores than eyes with glaucoma that had VF defects or eyes with glaucoma that did not have VF defects in both the visual and non-visual symptom domains of the GSS instrument. A significant inverse relationship between VF stage and both the visual and non-visual symptom scores of the GSS tool was seen after adjustment for age and BCVA.
