# Author Correction: Visual field loss and vision-related quality of life in the Italian Primary Open Angle Glaucoma Study

**DOI:** 10.1038/s41598-020-59555-6

**Published:** 2020-02-21

**Authors:** Eliana Rulli, Luciano Quaranta, Ivano Riva, Davide Poli, Lital Hollander, Fabio Galli, Andreas Katsanos, Francesco Oddone, Valter Torri, Robert N. Weinreb, Italian Study Group on QoL in Glaucoma, L. Varano, T. Carchedi, S. Talarico, P. Frezzotti, F. Parravano, I. Motolese, S. A. Bagaglia, G. C. M. Rossi, S. Lateri, L. Bossolesi, L. Carmassi, T. Rolle, R. Piccini, R. Ratiglia, A. Rossi, S. Gandolfi, V. Tagliavini, N. Ungaro, M. Fossarello, A. Cucca, I. Zucca, M. Uva, E. Bonacci, G. Cardarella, D. Tognetto, O. Vattovani, P. Vallon, F. Iannacone, L. Fontana, S. Marchi, G. L. Manni, D. Jannetta, G. Roberti, L. Rossetti, E. Maggiolo, O. Oneta, C. Sborgia, F. Cantatore, L. Mastropasqua, L. Agnifili, E. Campos, C. Gizzi, G. Giannaccare, V. Pucci, M. Cassamali, C. Costagliola, C. Traverso, R. Scotto, M. Musolino, L. Landi, A. Bagnis

**Affiliations:** 1 IRCCS - Istituto di Ricerche Farmacologiche Mario Negri, Milan, Italy; 2 DMSS, University of Brescia, Brescia, Italy; 3 IRCCS - Fondazione GB Bietti per lo Studio e la Ricerca in Oftalmologia, Rome, Italy; 4 Hamilton Glaucoma Center, Shiley Eye Institute, and the Department of Ophthalmology, University of California, San Diego, CA USA; 5 Università degli Studi “Magna Graecia”, Catanzaro, Italy; 6 A.O.U. Senese Ospedale Santa Maria delle Scotte, Università di Siena, Siena, Italy; 7 Fondazione IRCCS Istituto di Ricovero e Cura a Carattere Scientifico Policlinico San Matteo, Pavia, Italy; 8 IRCCS Istituto di Ricovero e Cura a Carattere Scientifico Istituto Auxologico Italiano, Milano, Italy; 9 Università degli Studi di Torino, Torino, Italy; 10 Fondazione IRCCS Istituto di Ricovero e Cura a Carattere Scientifico Ca’ Granda Ospedale Maggiore Policlinico, Milano, Italy; 11 A.O.U. di Parma, Parma, Italy; 12 A.O.U. Cagliari - Ospedale Civile San Giovanni di Dio, Cagliari, Italy; 13 A.O.U. “Policlinico Vittorio Emanuele” P.O. Gaspare Rodolico, Catania, Italy; 14 A.O.U. “Ospedale Maggiore”, Trieste, Italy; 15 A.O. Arcispedale Santa Maria Nuova-IRCCS, Reggio Emilia, Italy; 16 Università Tor Vergata, Fondazione Policlinico Tor Vergata, Roma, Italy; 17 A.O. S. Paolo, Milano, Italy; 18 A.O.U. Policlinico, Bari, Italy; 19 Ospedale Clinicizzato SS. Annunziata, Chieti, Italy; 20 A.O.U. Policlinico S. Orsola Malpighi, Bologna, Italy; 21 A.O. di Desenzano del Garda, Desenzano del Garda, Brescia, Italy; 22 Dipartimento SPES, Università del Molise, Campobasso, Italy; 23 IRCCS AOU San Martino - IST, Genova, Italy

Correction to: *Scientific Reports* 10.1038/s41598-017-19113-z, published online 12 January 2018

Paolo Frezzotti was omitted from the Italian Study Group on QoL in Glaucoma in the original version of this Article. This has been corrected in the PDF and HTML versions of the Article.

The Italian Study Group on QoL in Glaucoma now reads as:

“L. Varano, T. Carchedi, S. Talarico, P. Frezzotti, F. Parravano, I. Motolese, S. A. Bagaglia, G. C. M. Rossi, S. Lateri, L. Bossolesi, L. Carmassi, T. Rolle, R. Piccini, R. Ratiglia, A. Rossi, S. Gandolfi, V. Tagliavini, N. Ungaro, M. Fossarello, A. Cucca, I. Zucca, M. Uva, E. Bonacci, G. Cardarella, D. Tognetto, O. Vattovani, P. Vallon, F. Iannacone, L. Fontana, S. Marchi, G. L. Manni, D. Jannetta, G. Roberti, L. Rossetti, E. Maggiolo, O. Oneta, C. Sborgia, F. Cantatore, L. Mastropasqua, L. Agnifili, E. Campos, C. Gizzi, G. Giannaccare, V. Pucci, M. Cassamali, C. Costagliola, C. Traverso, R. Scotto, M. Musolino, L. Landi & A. Bagnis”

